# Development of Antimicrobial Paper Coatings Containing Bacteriophages and Silver Nanoparticles for Control of Foodborne Pathogens

**DOI:** 10.3390/v14112478

**Published:** 2022-11-09

**Authors:** Thanh Tung Lai, Thi Thanh Ha Pham, Marijn van Lingen, Gabrielle Desaulniers, Guy Njamen, Balázs Tolnai, Tarik Jabrane, Sylvain Moineau, Simon Barnabé

**Affiliations:** 1 Institut d’Innovations Écomatériaux, Écoproduits et Écoénergies à Base de Biomasse, Université du Québec à Trois-Rivières (I2E3, UQTR), Trois-Rivières, QC G8Z 4M3, Canada; 2 Kruger Inc., Montréal, QC H3S 1G5, Canada; 3 Innofibre—Centre d’Innovation des Produits Cellulosiques, Cégep de Trois-Rivières, Trois-Rivières, QC G9A 5E6, Canada; 4 Département de Biochimie, de Microbiologie et de Bio-Informatique, Faculté des Sciences et de Génie, Université Laval, Québec, QC G1V 0A6, Canada

**Keywords:** antimicrobial activity, bacteriophages, *Listeria monocytogenes*, paper coating, silver nanoparticles

## Abstract

In this study, a novel antimicrobial formula that incorporates Listeria bacteriophage P100 and silver nanoparticles into an alginate matrix was successfully developed. Paper coated with the antimicrobial formula inhibited the growth of Listeria monocytogenes. The effects of alginate concentration on the formation of silver nanoparticles, silver concentration on the infectivity of phages, and of low alginate concentrations on the sustained release of silver and phages were explored. The highest antimicrobial activity of the alginate–silver coating was achieved with an alginate concentration of 1%. Adding phage P100 (10^9^ PFU/mL) into the alginate–silver coating led to a synergic effect that resulted in a 5-log reduction in *L. monocytogenes*. A bioactive paper was then developed by coating a base paper with the antimicrobial formula at different coating weights, followed by infrared drying. The higher coating weight was a crucial factor for the maintenance of phage infectivity throughout the coating and drying processes. Phages incorporated into the alginate matrix remained functional even after high-temperature infrared drying. Taken together, an optimized coating matrix is critical in improving the antimicrobial performance of bioactive paper as well as maintaining phage infectivity during the paper manufacturing process.

## 1. Introduction

According to the Global Food Security Index, food quality and safety are among the four criteria for food security. In order to meet these criteria, the food industry continues to seek out advanced packaging technologies that improve quality and extend the shelf-life of food. In addition to acting as a physical barrier, packaging materials can possess preservative properties that protect the food from microbial spoilage and foodborne bacterial contamination [1]. Using as few chemical products as possible is also considered to be a crucial factor for food packaging. Thus, new active packaging systems using biobased materials have emerged, with bioactive surfaces consisting of antimicrobial substances. These new systems are a promising solution for meeting the above demands [2].

The use of paper-based packaging materials is a sustainable approach to food quality and safety. Paper is biodegradable, renewable and highly compatible with bioactive agents [3]. To use paper as a packaging material for food, specific treatment is required to improve the mechanical and barrier properties as well as the antimicrobial activity of the paper surface [4]. Paper coated with a polymer is used to create a functional paper surface [5,6,7]. For active paper packaging in which antimicrobial agents are incorporated, polymers are used as protectors as well as a delivery device for the antimicrobials. Indeed, the polymer matrix protects the antimicrobials from extreme conditions during the production and handling of the packaging materials, and also allows for a controlled, progressive release of antimicrobials, leading to effective and long-term antimicrobial activity for the packaged food [8,9,10]. Current research trends are moving toward natural and biodegradable sources, supporting the use of biopolymers as functional coatings due to their film-forming properties and biodegradability [11].

Bacteriophages (also called phages) are being increasingly explored as antibacterial agents due to their ubiquity, high host specificity and their ability to self-replicate [12,13]. The use of phages for the biocontrol of foodborne pathogens has garnered great interest during a time of growing concern for antimicrobial resistance in agri-food production [14,15]. Commercially available phage preparations are now available, and their efficacy has increasingly been documented [16]. Virulent phages are usually used for foodborne pathogen biocontrol, as they often readily lyse the targeted bacterial host cells, resulting in cell destruction and the release of a large number of progeny phages, which are then able to infect neighbouring targeted cells [12,13]. Dipping, spraying or even adding phage solutions directly to food are strategies proposed for food applications. However, these direct application methods often require large numbers of phages to be effective. These issues can be addressed by developing support materials on which phages are immobilized before their controlled release and interaction with the food [15]. The use of paper as the support material to immobilize phages has been successful in some studies [3,17,18].

Another antimicrobial agent that has been used in active food packaging is silver nanoparticles, which boast a broad spectrum of antimicrobial activity against foodborne pathogens. Certain works on risk assessment of potential toxicity of silver nanoparticles via migration as well as in vivo toxicity studies have shown a low level of risk from exposure to silver nanoparticle-containing packaging materials. The incorporation of silver nanoparticles into food contact materials to improve packaging properties has been exploited in the food industry [19,20]. The large surface of silver nanoparticles maximizes their contact with the microorganisms, allowing for cell permeation, denaturation and death [21]. One of the most useful methods for introducing silver nanoparticles to food packaging materials is to incorporate them into a biopolymer matrix for coatings. Some biopolymers, such as starch, chitosan, alginate, and gelatin, have been successfully mixed with silver nanoparticles, leading to high bioactivity [21].

Manufacturing bioactive paper by coating paper with antimicrobial agents would allow the pulp and paper industry to develop a new chain of products with no or minor equipment modifications [3]. However, one challenge is that the paper processing conditions can affect the stability and activity of the bioactive agents, resulting in a loss of functionality. Bioactive agents such as phages may be protected and immobilized through specific bioactive surface design and tailor-made coating formulas, in order to assure an efficient bioactive paper surface.

This study focuses on the development of a new antimicrobial coating for active paper-based food packaging. Coating formulas incorporate phages and silver nanoparticles into a biopolymer matrix. The concentration of ingredients in the coating formula was optimized to obtain maximum antimicrobial performance. The effect of paper coating and drying processes on the antimicrobial performance of bioactive paper against *L. monocytogenes* was also investigated.

## 2. Materials and Methods

### 2.1. Bacterial and Chemical Materials

*Listeria monocytogenes* serotype 4b strain (ATCC 19115) purchased from Cedarlane (Burlington, ON, Canada) was used as the bacterial host. A fresh bacterial culture was prepared by inoculating 300 µL of a stock culture in 2.7 mL of tryptic soy broth (TSB) and incubating at 30 °C for 18 h when the cells reached a concentration of approximately 10^9^ Colony Forming Units (CFU)/mL. The commercial phage product ListexTM P100 (Micreos, Wageningen, the Netherlands), containing phage P100 at a titer of approximately 10^11^ PFU (Plaque Forming Unit)/mL, was purchased from Think Ingredients (Burlington, ON, Canada) and was stored at 4 °C until use.

Chemical products including AgNO_3_ solution, phosphate buffered saline (PBS) and biopolymers including alginic acid sodium salt from brown algae, carboxymethylcellulose sodium salt (CMC) and gelatin from bovine skin type B gelatin (ACS grade), were purchased from Sigma-Aldrich (Oakville, ON, Canada). Culture media Tryptic soy agar (TSA), Tryptic soy broth (TSB) and agar were purchased from Difco (Montréal, QC, Canada) and *Listeria*-selective Oxford agar from Sigma-Aldrich (Canada). Microfibrillated cellulose (MFC) and the base papers with a basis of 50 g/m^2^ was kindly provided by Kruger Inc., Montréal, QC, Canada.

### 2.2. Preparation of Coating Solutions

Coating solutions were prepared by dissolving 1% (*w*/*v*) of each biopolymer in distilled water and stirring the solutions on a magnetic hotplate stirrer at 350 rpm and 60 °C. When homogenized solutions of biopolymers formed, the AgNO_3_ solution was gradually added to get final concentrations that varied from 0.05 to 14.5 mg/mL. The biopolymer–silver solutions were mixed vigorously at 60 °C without exposure to light for 1 h. The final solutions were stored in the dark at 4 °C for up to 2 weeks. Alginate–silver–phage coatings were prepared by homogenizing the alginate–silver solution by continuous agitation at 350 rpm, and then maintained at 25–30 °C. Phage P100 solution was gradually added during agitation until a final concentration of approximately 10^9^ PFU/mL was reached. The final solution was used immediately or stored at 4 °C in the dark for no longer than 24 h.

### 2.3. Lab-Scale Paper Coating

**Figure 1:**
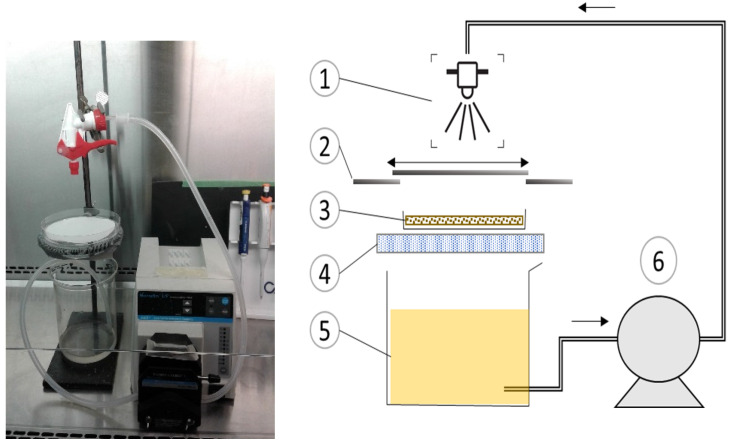
Customized spraying system. (1) 0.9-mm spray bottle nozzle, (2) customized shutter system, (3) 90-mm paper circle, (4) metallic 50-mesh screen, (5) antimicrobial formula, and (6) Masterflex peristaltic pump.

Base paper was cut to 6-mm or 90-mm diameter circles depending on the experiments. Prior to coating, both sides of the paper circles were sterilized by exposure to UV in a laminar flow hood for 30 min [22]. For the coating ingredient optimization experiment, an aliquot of 5 to 10 µL of coating solution was applied on the surface of the 6-mm paper sheets using the dropping pipette technique. The coated paper was air dried at ambient temperature for 40 min before further analysis. Bioactive papers were made by coating the 90-mm paper circles with the alginate–silver–phage solution using a customized spraying system (Figure 1). The spraying system consisted of a mesh, a 150-mm diameter glass dish placed underneath a 0.9-mm spray nozzle and a glass shutter. A peristaltic pump (Masterflex) was used to pump the formula from a reservoir to the spraying system. Spraying was repeated to obtain different coating weights. A glass stick was used as a metering blade to remove excess coating solution and to uniformly distribute the formula over the paper surface. The coated paper was then dried before further analysis.

### 2.4. Infrared Drying Process

The paper was dried using a 250 W infrared (IR) brooder lamp system. The coated paper was exposed to IR light at a distance of 30 cm for 90 s. The thermal profile of the paper surface was recorded using a FLIR T1030sc thermal imaging camera. The temperature of the paper surface was captured at a rate of 30 frames per second using FLIR software version 5.13.18031.2002(Wilsonville, OR, USA).

### 2.5. Transmission Electron Microscopy

The distribution of phage particles and silver nanoparticles (AgNPs) in the alginate polymer network and their particle sizes were analyzed using a transmission electron microscope (TEM EM208S, Philips) at 100 kV, which was equipped with a digital camera. One drop of the alginate–silver–phage solution was applied onto a copper grid covered by an amorphous carbon film and stained with 1.5% wt. uranyl acetate solution. The surface images of the samples were observed at magnifications of 36,000× to 70,000×. The average sizes of phage particles and AgNPs were determined by measuring a minimum of 100 particles using an ImageJ 1.53t (Wayne Rasband and contributors, National Institutes of Health, 10 Center Dr, Bethesda, MD 20814, United States. http://imagej.nih.gov/ij (accessed on 25 September 2022)) software package.

### 2.6. Antimicrobial Activity Assay

The antimicrobial activity against *Listeria* of the coating solutions and the coated paper were evaluated using the double layer agar (DLA) technique [23] and diffusion in liquid medium [24]. For the DLA method, a culture of *L. monocytogenes* was mixed into a soft TSA medium (0.75% agar) and poured on top of a solid TSA medium (1.5% agar) in a 90-mm plate for a final concentration of 5 × 10^5^ CFU/cm^2^. The DLA plates inoculated with *L. monocytogenes* were allowed to settle for 10 min before adding an aliquot (5–10 µL) of coating solution or placing the 6-mm paper circle samples on the agar layer surface. The DLA plates were incubated at 30 °C for 72 h. The antimicrobial activity was determined by measuring lysis areas in lawns of *L. monocytogenes* at specific time intervals during the incubation.

### 2.7. Phage Enumeration

Phage titers in the coating formula or loaded onto coated paper were determined using the DLA technique. For the coated paper, phages were released by submerging 6-mm paper circles in 15-mL tubes containing 5 mL of PBS buffer, which were then agitated at 120 rpm at 30 °C. Suspension samples were collected at 24-h intervals for the DLA method for 72 h. The phage titer was expressed as plaque-forming units per cm^2^ (PFU/cm^2^) of paper. Experiments were performed in triplicate.

## 3. Results and Discussions

### 3.1. Silver-Incorporated Biopolymer Coating

At first, the paper coatings by different biopolymers supplemented with silver were studied. The impact of biopolymers on silver nanoparticle formation and on the antimicrobial activity against *L. monocytogenes* were assessed.

#### 3.1.1. Selection of Biopolymers

**Figure 2:**
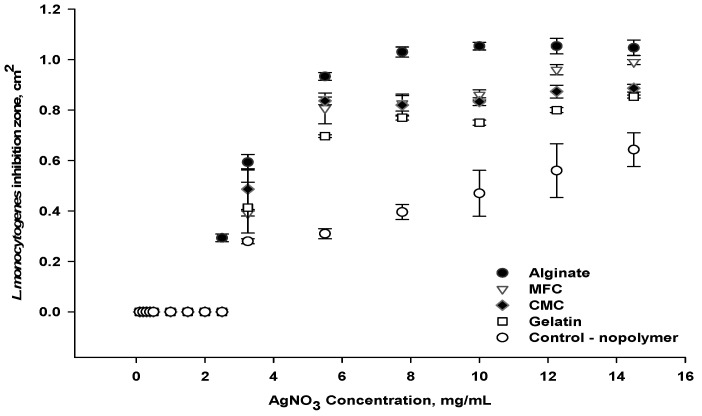
Antimicrobial activity of coated paper using silver and different polymers.

Polysaccharide polymers, alginate, microfibrillated cellulose (MFC), carboxymethyl cellulose (CMC) and a protein-based polymer in the form of gelatin were each blended with AgNO_3_ to produce a coating solution. The antimicrobial activity against *L. monocytogenes,* determined by measuring the area of lysis in the DLA assay after 24 h of treatment, are shown in Figure 2. First, we confirmed that these polymers (without antimicrobials) do not inhibit the growth of *L. monocytogenes* by the DLA method. The paper coated with the polymer–silver blend exhibited a stronger antimicrobial effect than the silver-coated paper, revealing an improvement in activity when the biopolymer is mixed with silver. The biopolymer matrices likely acted as reducing and stabilizing agents which led to active silver nanoparticles (AgNPs) from the AgNO_3_ [21].

The biopolymers also likely retain active AgNPs in their three-dimensional polymeric network without aggregation, resulting in a controlled release of AgNPs from the polymer matrix into the surrounding medium [19,25]. In addition, the interface of the cellulosic fibers of the paper with the polymer–silver matrix led to a porous structure on the coated paper, which likely facilitated water adsorption, thereby releasing AgNPs more efficiently [20,26].

The antimicrobial activity also increased with the AgNO_3_ concentrations for all polymer–silver coatings, suggesting that the AgNPs are easily incorporated and well-dispersed within the polymer matrix. The combination of alginate and silver resulted in the best antimicrobial performance against *L. monocytogenes*, followed by the combinations of silver with MFC, CMC and gelatin, respectively. Increasing the AgNO_3_ concentration from 1 to 8 mg/mL improved the antimicrobial activity of paper coated with the alginate–silver blend, as demonstrated by the larger area of *L. monocytogenes* growth inhibition on the DLA from 0.28 ± 0.01 to 1.0 ± 0.05 cm^2^. The highest activity obtained with the alginate–silver blend could be attributed to the interaction of alginate and silver nanoparticles. According to previous studies, the addition of silver ions (Ag^+^) to the alginate solution served as a cross-linking agent for the formation of an alginate hydrogel, which supported AgNPs formation [22,27]. Others showed that the hydrophilic alginate hydrogel in turn facilitated the release of AgNPs from the polymeric network, which likely resulted here in inhibiting of *L. monocytogenes* [28]. An alginate polymer was selected for further exploration of antimicrobial coating formulas.

#### 3.1.2. Effect of Alginate Concentration

**Figure 3:**
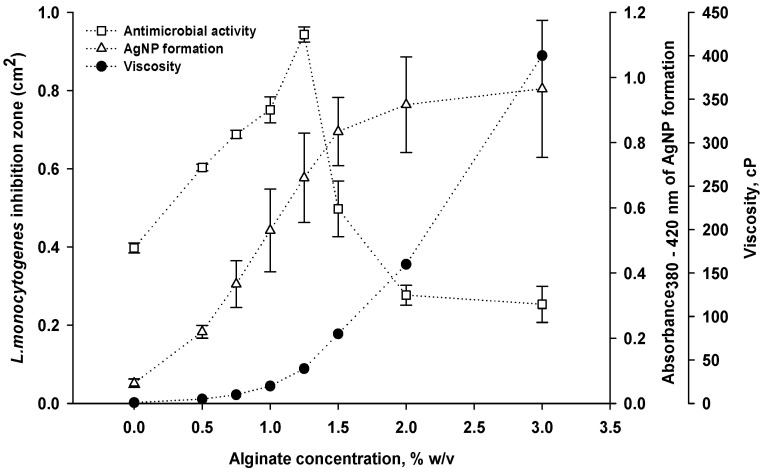
Effect of alginate concentration on antimicrobial activity, viscosity and AgNPs formation of alginate–silver coating.

Coating formulas that combined 5.5 mg/mL of AgNO_3_ with concentrations of alginate ranging from 0.5 to 3% *w*/*v* were tested. The varying alginate concentrations in the coating solutions resulted in changes in viscosity, AgNPs formation and antimicrobial activity against *L. monocytogenes* (Figure 3). According to previous studies, the most typical evidence of AgNPs formation is the appearance of a strong surface plasmon resonance band that is observable in the region of 350–600 nm [20,28].

As shown in Figure 3, the absorbance (380–420 nm) of our coating solutions increased as a function of the alginate concentration, reaching a maximal absorbance of 1.0 at an alginate concentration of 3.0%. It suggests that the formation of AgNPs is improved when the concentration of alginate increases.

However, antimicrobial activity reached a maximum inhibition (area of 0.92 ± 0.07 cm^2^) with an alginate concentration of 1.25% *w*/*v,* but decreased (0.26 ± 0.05 cm^2^) when alginate was increased to 3.0% *w*/*v*. The proportional increase in coating solution viscosity with alginate concentration (shown in Figure 3) likely resulted in an aggregation of AgNPs, obstructing the contact between AgNPs and bacteria [20]. Others [29] reported that a cellulosic surface is thoroughly covered by the micro- or nanostructure polymer–ZnO coating at coating weights of 3 g/m^2^. Increasing the coating weight to 4.5 g/m^2^ resulted in densely packed layers of polymer–ZnO coating on the cellulosic surface, leading to a significant loss in micro- or nano hierarchical structures that reduced antimicrobial efficiency. This outcome is consistent with our results, in which an alginate concentration range of 1.0–1.25% that corresponded to dried paper coating weights of 1.8–2.3 g/m^2^ were the most effective for antimicrobial activity against *L. monocytogenes*. Because coating weights clearly affect the antimicrobial efficiency, adjustments should be considered during the coating formula application process. This is especially true for spray coating, which might require a high coating weight.

### 3.2. Incorporating Phages into Alginate–Silver Coating

In order to design an antimicrobial coating formula containing phages, as a proof of concept we incorporated the commercially available *Listeria*-specific phage P100 into the alginate–silver matrix. It was expected that the addition of phages into the alginate–silver coating would provide a synergistic effect for the control of *L. monocytogenes*. Using alginate hydrogel as the delivery system would allow for a sustained release of the phages and AgNPs to prolong the duration of treatment.

#### 3.2.1. Effect of Silver Concentration on Phage Infectivity

**Figure 4:**
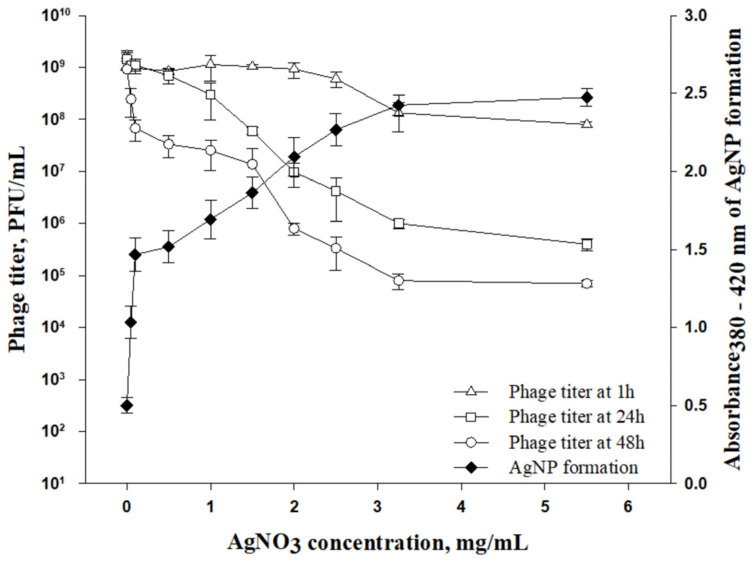
Effect of AgNO_3_ concentration and incubation time on infectivity of phages incorporated in alginate–silver coating.

When exposing phages to the alginate–silver matrix, the AgNPs may affect phage infectivity. Previous studies on phage–silver interaction have demonstrated that the sensitivity of phages to metallic nanoparticles depends on the concentration and size of the nanoparticles [30,31]. Different coating formulas containing 0.5% alginate and AgNO_3_, at concentrations that ranged from 0.05 to 5.5 mg/mL, were used to incorporate phages at a concentration of 10^9^ PFU/mL. The coating formulas were stored in the dark at 4 °C and phage titers were estimated after 1 h, 24 h and 48 h. As shown in Figure 4, the phage titer was reduced proportionally with increases in AgNO_3_ concentrations from 0.5 to 3 mg/mL. When AgNO_3_ was at its highest concentration of 5.5 mg/mL, phage titers were significantly reduced by 3.5 and 4.5 log after 24 h and 48 h, respectively. Meanwhile, there was a reduction of only 1 log unit in the phage titer with the coating formula sampled after 1 h. According to spectrophotometric measurements at 380–420 nm, the absorbance of the alginate–silver solution increased from 1.5 to 2.4, along with an increase in AgNO_3_ concentration from 0.5 to 3 mg/mL, resulting in increased AgNPs formation (Figure 4).

This might indicate that the phages were inactivated when certain levels of AgNPs were reached, which were determined by the initial AgNO_3_ concentration and storage time. At low AgNO_3_ concentrations, ranging from 0.05 to 0.45 mg/mL, the reduction in phage titers was not significant (0 to 1.5 log-unit reductions) as a function of storage time (from 1 h to 48 h). AgNP absorbance was approximately 1.0 at these low AgNO_3_ concentrations, meaning that there was adequate AgNP formation for antimicrobial efficiency. Thus, for the preparation of the coating formula, the concentration of AgNO_3_ should remain lower than 0.45 mg/mL in order to minimize negative effects on the phages.

#### 3.2.2. Effect of Phage Dose on *L. monocytogenes* Growth Inhibition

**Figure 5:**
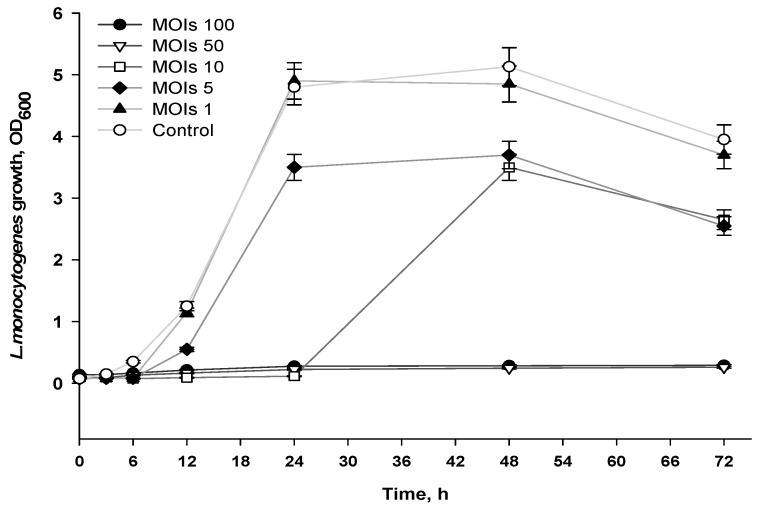
Antimicrobial activity of formulas applied at different MOIs.

Formulas that consisted of 0.5% alginate, 0.05 mg/mL AgNO_3_ and phages at titers ranging from 10^5^–10^7^ PFU/mL were added to approximately 10^5^ CFU/mL of *L*. *monocytogenes*, resulting in ratios of phages to bacteria (multiplicity of infection, MOIs) of 1, 5, 10, 50 or 100 at the time of infection. The formulas containing cultures were then incubated for 72 h and the optical density was measured at different intervals. As shown in Figure 5, MOIs of 50 and 100 prevented *L. monocytogenes* growth (OD_600 mm_ ≤ 0.1) over 72 h. An MOI of 10 prevented *L. monocytogenes* growth for only 24 h.

At lower MOIs (1, 5 and 10), *L. monocytogenes* continued to grow, with OD_600 mm_ values ranging from 3.3 to 4.7 after 72 h compared to non-treated *L. monocytogenes* culture, which had an OD_600 nm_ of 5.2. These observations are consistent with previous research that demonstrated that higher phage concentrations are more effective in inhibiting bacterial growth [18,32]. Thus, it is important to consider the use a sufficient quantity of phages in coatings to prevent bacterial growth, which would lead to extending the shelf-life of food.

### 3.3. TEM Analysis of Alginate/AgNPs/Phage Formula

**Figure 6:**
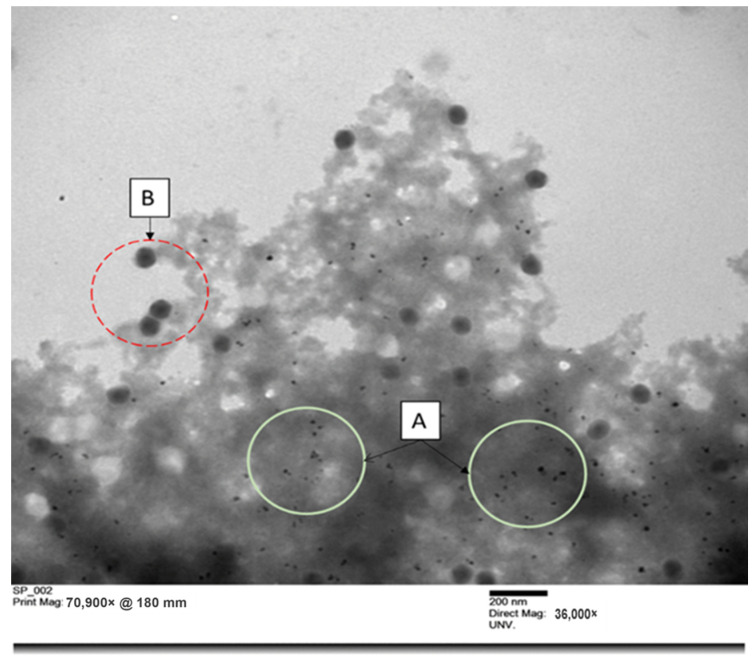
Distribution of phage particles and silver nanoparticles in alginate matrix observed using transmission electronic microscope: (A) AgNP particles, (B) phage particles.

The phage–AgNPs–alginate matrix was visualized under a transmission electron microscope (TEM) (Figure 6). Phage particles and AgNPs had a homogeneous distribution in the alginate network and confirmed the presence of AgNPs in spheroid form. The AgNPs were 10–20 nanometers in diameter, whereas the capsid (90 nm) of phage P100 could be easily distinguished in the polymer matrix [33]. Interestingly, the phage and AgNPs appeared to remain separate from each other, which could reduce the negative impact of AgNPs on phages.

### 3.4. Lab-Scale Fabrication of Bioactive Paper

The alginate–silver-phage (AAP100) coating formula, at optimized concentrations (10^9^ CFU/mL phages, 0.05 mg/mL AgNO_3_ in 0.5% alginate solution), was used to produce a bioactive paper. Coating experiments were conducted using a laboratory spraying system and paper circles that were 90 mm in diameter. The AAP100 formula was applied to the paper at different coating weights: 16, 31, 47, 63, 79, 94 and 110 g/m^2^. The coated paper circles were then dried under IR lamps for 90 s or air-dried for 40 min. The effect of the drying method along with the coating weights on antimicrobial performance was then investigated.

**Figure 7:**
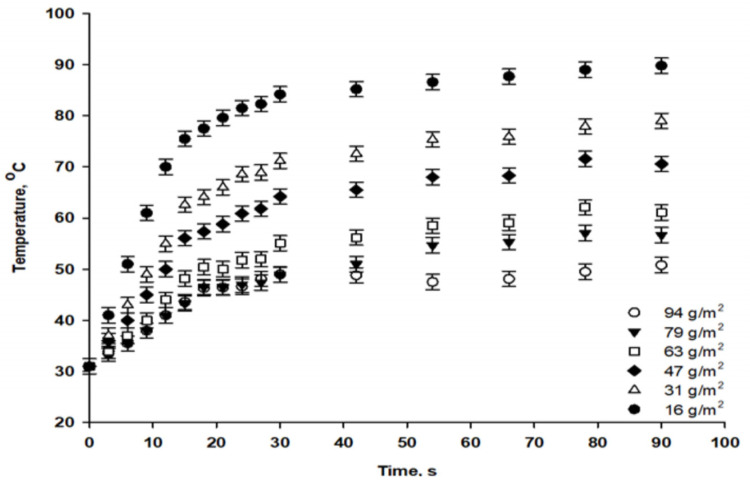
Temperature profile detected on the surface of coated papers at different coating weights (g/m^2^) during infrared drying.

Figure 7 shows the temperature profiles of the paper circles during IR drying, recorded with a camera (FLIR T1030sc IR). Paper coated at low coating weights reached higher temperatures more quickly. After 90 s of drying, the highest temperatures reached were 90 °C for a coating weight of 16 g/m^2^ and 50 °C for a coating weight of 94 g/m^2^. In industrial paper production, the IR drying technique is often used in binder-coating processes to enhance coating uniformity and improve overall paper quality [34]. For a dry end of coated paper, the temperature should reach 90 °C to ensure moisture removal, as was observed for paper with a coating weight of 16 g/m^2^.

**Figure 8:**
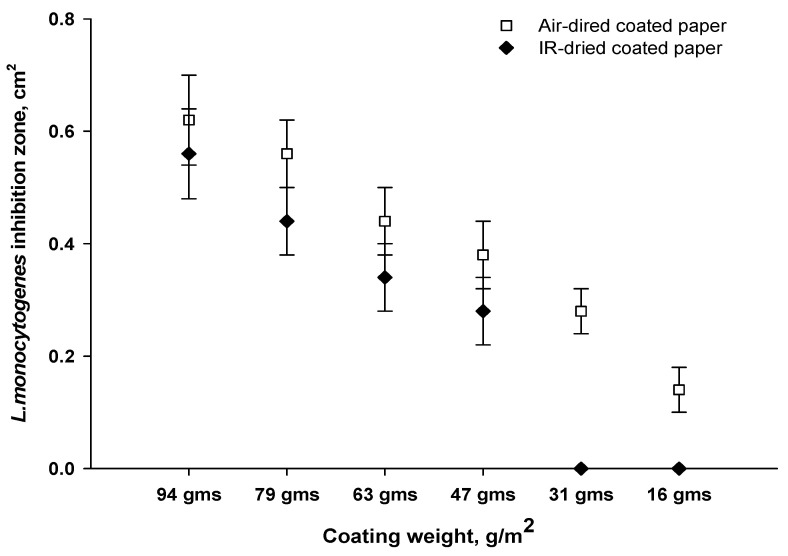
Effect of coating weight and drying on antimicrobial performance of bioactive papers.

The antimicrobial performance of IR-dried paper (Figure 8) revealed a decrease in the lysis zone with a decrease in coating weight. There was no antimicrobial activity with the IR-dried paper at coating weights of 16 g/m^2^ and 31 g/m^2^. For the air-dried paper, antimicrobial activity also decreased with decreasing coating weights, but antimicrobial efficiency was still observed with the lowest coating weight of 16 g/m^2^. Our data clearly indicated that the IR-drying temperatures affected the antimicrobial efficiency of the coated paper. The structure and properties of the coated paper were influenced by high-temperature drying, which induced a dried coating structure and caused poor antimicrobial activity [35].

**Figure 9:**
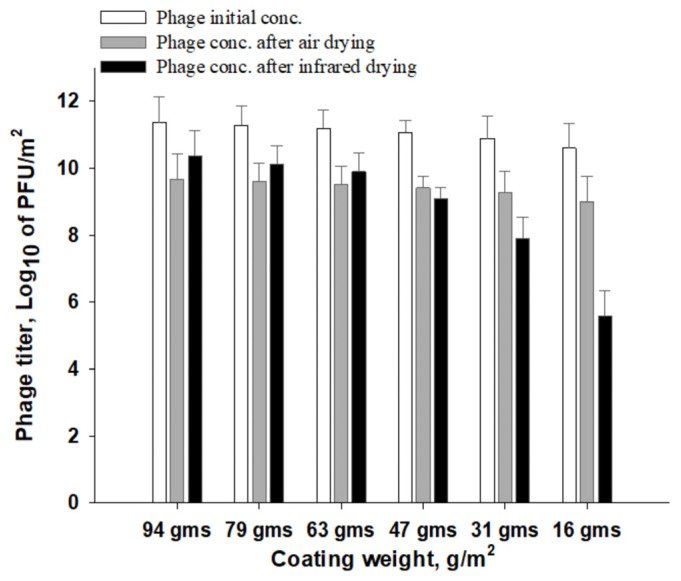
Effect of coating weight and drying method on phage infectivity as a result of initial phage concentration (white), phage concentration after air-drying (gray) and phage concentration after IR-drying (black).

The number of phages that remained infectious on IR-dried paper was compared to that which was initially added to the formula (Figure 9), revealing about a 1-log titer reduction for coating weights from 63 to 94 g/m^2^. Larger phage reductions of 2, 3.7 and 5-log units were observed for IR-dried papers at coating weights of 47, 31 and 16 g/m^2^, respectively. Phage P100 was more stable in air-dried paper, with a reduction of about 1.5-log phage titer for all coating weights. Based on antimicrobial activity and phage infectivity, we conclude that the high temperatures of IR drying negatively affect phage stability. Increasing the coating weight improved the stability of the phages under extreme IR-drying conditions, which can be attributed to the protective effect of the alginate–silver matrix on the phages during heat treatment. In addition, at high coating weights, the coating structure was less influenced by IR drying, allowing phages to remain dispersed in the alginate network and to be released during antimicrobial experiments. Phages can be sensitive to dehydration and heat treatment [36,37,38]. As observed in the thermal profile (Figure 8), the temperature reached 50 °C for high coating weights, leading to low levels of water evaporation from the coating layer. This may have allowed the coated paper to retain the moisture necessary for phage stability. In contrast, at low coating weights, coating layer dehydration and consolidation associated with high IR temperatures could have led to the penetration of phages from the coating layer into the base paper, resulting in a poor release of phages during the test.

For large-scale manufacturing of bioactive paper, IR drying is a practical approach into the paper-making process. Thus, the control and optimization of IR-drying temperatures and coating weights is critical for the maintenance of phage activity and the antimicrobial performance of treated paper. For example, IR-dried paper that lacks moisture could undergo a rewetting step, which may also improve the diffusion of phages that are trapped in the paper matrix [36]. Phages could also be applied at high concentrations into the coating formula in order to increase the antimicrobial efficiency of phage-based bioactive paper [18] and progress has been made in the production of *Listeria* phages [23].

## 4. Conclusions

Overall, our results showed that the addition of phages and silver nanoparticles into an alginate matrix can provide antimicrobial properties to paper through coating techniques. Indeed, a bioactive paper was successfully developed using an antimicrobial formula based on alginate supplemented silver nanoparticles and a commercial phage preparation. The combination of phages and silver nanoparticles was demonstrated to be effective in limiting the growth of *Listeria*. The phages and the silver nanoparticles were well-distributed in the alginate network, leading to a sustained release of these particles in our laboratory tests. A high coating weight of the phage-based formula is required for maintenance of phage activity when undergoing high-temperature infrared drying. These methods can be used by the pulp and paper industry to produce antimicrobial paper-based food packaging. Further research is needed on the antimicrobial efficiency of bioactive paper for in vivo *Listeria* control tests on targeted foods and the shelf-life of packaged food. In addition, it remains to be seen how other phages will behave in this coating formula.

## Data Availability

Not applicable.
